# The Red Cross Society

**Published:** 1914-08-29

**Authors:** 


### The Red Cross Society.

### The Red Cross Expedition to Belgium.

The Red Cross Society have sent out to Belgium an expedition consisting of ten surgeons, ten dressers, and twenty fully trained hospital nurses, who left Charing Cross under the command of Major the Hon. Robert White. Dr. Wyatt was in command of the surgeons. The Society also sent out Major Richardson with his Red Cross dogs (bloodhounds), who was to take with him Mr. Cherry Garrard, of Captain Scott's expedition, whose exploits are well known. This Belgian expedition is in charge of Sir Alfred Keogh, and it includes a number of notable men, who are going with him on duty with the Red Cross forces in Belgium. Sir Frederick Treves, Bart., G.C.V.O., C.B., announced last week that Red Cross units will be leaving for the front practically every day. A unit consists of ten surgeons, ten dressers, and twenty fully qualified hospital nurses.

### The Red Cross Motor-car Corps.

A Motor Corps has been formed for Red Cross work in Great Britain. The corps is under the supervision of Lord Norreys and Sir Frederick Treves, who are eager to hear from owners willing to place their cars at the disposal of the British Red Cross Society, with or without drivers. The cars will be used for carrying patients and stores. Owners should address Dept. No. 8, the British Red Cross Society, Devonshire House, Piccadilly. The total of subscriptions received already approaches £40,000.

### Progress of the Arrangements for Stores.

Owing to the large amount of garments and stores of various kinds which are being received by the British Red Cross Society, it has been found necessary to secure more accommodation. All garments and stores of all kinds should, therefore, be sent, not to Devonshire House, but to the Stores Department, British Red Cross Society, Pall Mall, London, S.W. In fact Mr. Rodman Wanamaker, the donor of the silver altar which was put up in Sandringham Church to the memory of the late King Edward, has placed the whole of his premises at 13 and 14 Pall Mall, and entire staff, at the disposal of this Society for use as the Stores Department. Owing to the great quantity of garments, medical necessities, etc., which are being received at Devonshire House, the accommodation there has been found insufficient. Ladies have been asked to enroll their names as members of Lady Garvagh's working parties, which are to be held daily at her house, 4 Marble Arch, to provide clothing for the wounded through the medium of the Red Cross Society. The garments are cut out by skilled dressmakers who have generously given their services.

"The Islanders" (Patron, H.R.H. the Princess Royal; Vice-President, Lord Grey; Chairman, Lord Esher) are establishing a branch for sewing centres in connection with the British Red Cross Society at Craig's Court House, their headquarters in Whitehall. Two sewing parties will take place simultaneously. The Singer Sewing-Machine Company have kindly lent to the Society a large number of their sewing-machines.

### THE WAR OFFICE AND PRIVATE OFFERS.

Sir Arthur Sloggett, K.C.B., Director-General of the Army Medical Corps, wishes it to be clearly understood that the War Office cannot undertake to utilise any hospital unless it is officially approved by the British Red Cross Society.

Mr. Mortimer Singer has offered the Red Cross Society the use of his newly built house, Milton Hill, Steventon, Berkshire, as a convalescent home for wounded soldiers and sailors. The hospital is fully equipped for the maintenance of 200 patients, and every expense in connection will be defrayed by Mr. and Mrs. Singer's personal generosity.

The British Red Cross Society has established Clevedon as a Convalescent Base. The local Voluntary Aid Detachments have offered to provide and equip a hospital of fifty beds, and Mr. E. S. Wills has offered "Oaklands," which will probably be used for this purpose.

Badminton has been established as a depôt for the hospital and general comforts department of the Gloucester branch of the Red Cross Society.

The Bishop of Bristol has placed the episcopal palace at the disposal of the Red Cross Society, and Mr. R. E. Bush, of Bristol, has fitted up his residence with 100 beds, for use if required.

The Temporary Convalescent Home Advisory Committee has joined with and is now worked under the British Red Cross Society by means of a Joint Sub-Committee.

### A LINK BETWEEN FIRING LINE AND HOSPITAL.

Before the outbreak of war the Voluntary Aid Detachments of the British Red Cross Society had a total of about 60,000 members, men and women, who had been in regular training, organised on the following basis:—

Men's Detachment.

1 commandant.
1 medical officer.
1 quartermaster.
1 pharmacist.
4 section leaders.
48 men (divisible into four sections of twelve men each).

Total... 56

Women's Detachment.

1 commandant (man or woman, and not necessarily a doctor).
1 medical officer (to be attached when available and when the commandant is not a doctor).
1 lady superintendent, who should be a trained nurse.
1 quartermaster (man or woman).
1 pharmacist.
20 women, of whom four are qualified as cooks.

Total... 25

In times of war the above detachments are mobilised as an integral part of the Territorial Forces. The Territorial Forces already have their Ambulance Corps and their base hospitals fully equipped. The Red Cross Detachments are the link between the two. The Ambulance Corps bring in the wounded from the firing line; the Red Cross Detachments provide such treatment as is immediately possible, and then, if necessary, pass them on to the base hospital for further treatment.

### HOW OFFERS OF HELP ARE DEALT WITH.

There are thousands of men and women asking how best to help throughout the country. Those who are anxious to offer personal service in the Red Cross cause should, if untrained, apply to the County or Borough Secretary of their own district. The Secretaries organise working parties for ladies, emergency classes, &c., and keep registers of employment for voluntary help of every kind. But even more urgent than the need for personal service is the need for money.

### A LADY-RESIDENT VOLUNTEERS.

Among those who have volunteered for service at the front is Dr. A. Grange Fergus, one of the lady assistant resident medical officers at St. Luke's Hospital, Bradford.

### RED CROSS WORK AT CAMBRIDGE.

*(From our Special Correspondent.)*

Both Red Cross and Voluntary Aid Detachments are very busy at Cambridge, as may be seen from the Borough Detachment's work. Taking the Women's Detachments, Cambs 8, 28, and 40, as an instance, the requisitioning is completed and the stores are packed. Pembroke College Hall has been equipped as a ward for the purpose of practice. Each detachment receives in the hall each week three drills. Patients for the purpose are provided by the Girl Guides. The commandant of each detachment in turn is responsible for arranging for three nurses to undertake duties at the Workhouse Infirmary each day. Arrangements have also been made whereby Red Cross nurses in turn attend daily at Addenbrooke's Hospital to witness surgical dressings, etc., being carried out, and for the more advanced members of the various detachments a course of lectures is being delivered by Mr. W. H. Bowen, M.S.Lond., F.R.C.S.Eng., each week in the Old Library at Pembroke College.

As to the men's detachments, Cambs 7 Detachment, the only one of its kind in the Borough of Cambridge registered before the outbreak of war, receives daily practice in stretcher drill, bandaging, and other useful branches of work. Squad E, attached formerly to Cambridge Detachment 8, has now been raised to full strength and has been registered as a new detachment, and it is expected will soon receive from the War Office its official number, in all probability "Cambs 13."

Two new detachments are in process of formation, the one drawn from employees of the Cambridge University Press, the other from university and college servants. Each of these detachments has at present between seventy and eighty members, and is divided for drill and lectures into half-detachments working separately. These receive in Pembroke College or at the headquarters, at the rear of Addenbrooke's Hospital, in Tennis Court Road, one first-aid lecture and three stretcher drills.

The Colonel-in-Chief, Dr. Joseph Griffiths, of the Territorial Force, attached to the first Eastern General Hospital, lately paid a surprise visit to Pembroke College. He inspected the ward and the work in which the members were then engaged, and expressed himself satisfied with the thorough way in which the duties were being carried out.

At Addenbrooke's Hospital at least sixty beds have been placed at the disposal of the War Office. From the many thousands of troops now encamped at Cambridge, seven or eight soldiers have been admitted for treatment at this hospital.

### A HOSPITAL UNIT AT GREENWICH

### FOR RECEIVING WOUNDED IN THE THAMES.

The *Dreadnought* Hospital at Greenwich has now established a complete unit for the treatment of sick and injured men of the Royal Navy who may arrive at any moment in the Thames from the Fleet. An arrangement has been made with the Admiralty for 200 beds in the first instance, and these are now fully equipped and ready for use. The honorary medical staff have offered their services to treat free of all charge any men who may be injured in the war at sea.

A particular feature of the arrangement at Greenwich is that in the immediate vicinity of the hospital there is ample opportunity to provide a very large number of additional beds, and should occasion require it these will be equipped with the least possible delay. There is no lack of the supply of nurses and other assistants, and there can be little doubt that should a large number of patients be suddenly put upon the hospital the resources of the establishment will be equal to the occasion.

It has been pointed out that no more appropriate place can be found for the landing of sick and injured than the pier at Greenwich, which is close to the hospital. Here patients can be removed direct from the vessel to the hospital beds without the distress of transference to ambulance or other conveyance. In this respect the hospital at Greenwich is in a unique position, no other establishment having exactly these facilities.

The *Dreadnought* has been for a number of years under reconstruction. Three separate grants have been made to the Society for this purpose by King Edward's Hospital Fund for London. First the west wing was reconstructed by the removal of the partition walls which existed between a number of small wards and by the re-flooring of that part of the building. This first alteration took place in the year 1908.

In 1910 a further grant from King Edward's Hospital Fund enabled the Somerset Wing, a detached building, to be somewhat similarly dealt with, and this year a further grant has enabled the medical side to be entirely remodelled, though the Society has had to dip fairly deeply into its reserves to make up the additional cost of bringing this, the oldest part of the building, up to modern requirements.

The old building contains some of the finest wards in London, and it is remarkable that an edifice erected in 1763 should have so readily adapted itself to what is generally needed in a modern London hospital. This wing is a portion of the reserve of beds for men of the Royal Navy, and will be the first to receive the sick and injured who will arrive in this country after the first naval engagement.

The following members of the honorary medical staff are now on active service: Major G. N. Biggs, 4th London R.A.M.C., Capt. R. E. Bickerton, 2nd London R.A.M.C., Lieut. E. W. Gandy, and H. B. Carlill. In addition to the arrangements made at Greenwich, twenty-five beds are placed at the disposal of the Admiralty at the Albert Dock Hospital. It is believed that no matter how many men may come from the Fleet it will not be necessary to deprive the men of the Mercantile Marine of the benefits of the hospital, for it is obvious to anyone that the merchant seaman runs almost as much risk from submarine mines and the dangers of the sea as his brother in the Royal Navy.

The war has also affected the two schools of medicine attached to the Seamen's Hospital Society. The London School of Tropical Medicine will open for the winter session as usual on October 1, but, instead of having between sixty and seventy students—the average number—it is not anticipated that more than twenty will be in attendance. The present conditions rob the school of all Indian Medical Service students, of the naval surgeons, and of many from the Colonial Medical Service. In addition to this, a proportion of the prospective students have offered their services to their country, so that those who attend will be principally private students, some American and foreign graduates, and a few missionary officers. The course in tropical sanitation and hygiene, advertised for the winter session, will not be held. As regards the London School of Clinical Medicine at Greenwich, which is so largely attended by surgeons of the Royal Navy, a public announcement as to whether the October session will be held will shortly be made.

### War Notes.

One of the curious developments of the war is found in the case of a prince, who is now in arms against the Allies, and whose agent has offered the prince's mansion in Sussex to the British Red Cross Society for the Society's use during the war.

The War Office has accepted an offer of the Wenlock Lady Forester Trust of twenty-five beds at their Much Wenlock Hospital and of twenty-five beds at their Broseley Hospital, as well as the loan of their Convalescent Home at Llandudno.

On Friday last week the British Red Cross Society announced that no more offers of accommodation were wanted. The greatest need is the need of more money. On the same date further stores of surgical supplies were sent to Belgium, and 100 mattresses and 100 bolsters followed last Saturday.

### OLD MASTERS AND THE WAR.

At the Grosvenor Gallery, New Bond Street, an exhibition of ancient and modern pictures has been opened in aid of the Red Cross Society. Works by Veronese, Dobson, Aert Van der Neer, Zoffany, Constable, Manet, Wilson, and paintings and drawings by Rossetti, Strang, Charles Shannon, William Greaves, Degas, and others are on view. The collection is open between 10 and 6, and the charge for admission is one shilling.

